# CMNPD: a comprehensive marine natural products database towards facilitating drug discovery from the ocean

**DOI:** 10.1093/nar/gkaa763

**Published:** 2020-09-28

**Authors:** Chuanyu Lyu, Tong Chen, Bo Qiang, Ningfeng Liu, Heyu Wang, Liangren Zhang, Zhenming Liu

**Affiliations:** State Key Laboratory of Natural and Biomimetic Drugs, School of Pharmaceutical Sciences, Peking University, Beijing 100191, China; National Resource Center for Chinese Materia Medica, China Academy of Chinese Medical Sciences, Beijing 100700, China; State Key Laboratory of Natural and Biomimetic Drugs, School of Pharmaceutical Sciences, Peking University, Beijing 100191, China; State Key Laboratory of Natural and Biomimetic Drugs, School of Pharmaceutical Sciences, Peking University, Beijing 100191, China; State Key Laboratory of Natural and Biomimetic Drugs, School of Pharmaceutical Sciences, Peking University, Beijing 100191, China; State Key Laboratory of Natural and Biomimetic Drugs, School of Pharmaceutical Sciences, Peking University, Beijing 100191, China; State Key Laboratory of Natural and Biomimetic Drugs, School of Pharmaceutical Sciences, Peking University, Beijing 100191, China

## Abstract

Marine organisms are expected to be an important source of inspiration for drug discovery after terrestrial plants and microorganisms. Despite the remarkable progress in the field of marine natural products (MNPs) chemistry, there are only a few open access databases dedicated to MNPs research. To meet the growing demand for mining and sharing for MNPs-related data resources, we developed CMNPD, a comprehensive marine natural products database based on manually curated data. CMNPD currently contains more than 31 000 chemical entities with various physicochemical and pharmacokinetic properties, standardized biological activity data, systematic taxonomy and geographical distribution of source organisms, and detailed literature citations. It is an integrated platform for structure dereplication (assessment of novelty) of (marine) natural products, discovery of lead compounds, data mining of structure-activity relationships and investigation of chemical ecology. Access is available through a user-friendly web interface at https://www.cmnpd.org. We are committed to providing a free data sharing platform for not only professional MNPs researchers but also the broader scientific community to facilitate drug discovery from the ocean.

## INTRODUCTION

Natural products and their molecular frameworks play a highly significant role in the drug discovery and development process. Approximately two-thirds of all small-molecule approved drugs from January 1981 to September 2019 owe their origins to natural products (1). As common and easily accessible resources for humans, terrestrial natural products have long been a traditional source of drug molecules. With the impressive progress of techniques for sample collection (e.g. scuba diving, deep-sea exploration), compound separation (e.g. HPLC) and structure determination (e.g. NMR spectroscopy, X-ray crystallography), marine natural products (MNPs) chemistry have gradually developed and attracted widespread attention (2). Approximately 70% of the Earth’s surface is covered by oceans, which host a wealth of unexplored biological resources. The ocean’s extreme variations in pressure, salinity, temperature, pH, availability of nutrients and light make the secondary metabolites of marine organisms present incredible diversity in both chemical space and biological activities (3). Over 30 000 MNPs have been discovered since the first report of biologically active MNP spongothymidine in 1950 (4). Marine innovative drug discovery has become a hotspot in global drug research and development.

Access to suitable databases is essential for the comprehensive research of MNPs, such as the discovery of new substances, the synthesis of known compounds or analogues, the analysis of taxonomic and geographic information of source organisms, and the study on bioactivities (5). Yet, there is still a small number of databases dedicated to MNPs research. The commercial databases MarinLit (http://pubs.rsc.org/marinlit) and Dictionary of Marine Natural Products (http://dmnp.chemnetbase.com) are currently the most exhaustive and complete MNPs databases, but subscription fees may prevent their broader access to academic research. The recently established academic free database MarinChem3D (http://mc3d.qnlm.ac) provides 3D structures of MNPs, but its biological activity data is limited. Some open access databases such as the Seaweed Metabolite Database (SWMD) (6) and the Dragon Exploration System on Marine Sponge Compounds Interactions (DESMSCI) (7) contain only natural products produced by certain types of marine organisms. Other MNPs-related databases are relatively small, and most of them have not been updated for a long time. Generic chemical databases such as Reaxys (https://www.reaxys.com), PubChem (8), ChEMBL (9) and ChemSpider (10) include a certain number of MNPs, but the lack of sufficient annotations makes it difficult to retrieve MNPs from tens of millions of compounds. There is still a need for a free and complete professional MNPs database.

Here, we present CMNPD, a comprehensive marine natural products database, which includes information on chemical entities with various physicochemical and pharmacokinetic properties, standardized biological activity data, systematic taxonomy and geographical distribution of source organisms, and detailed literature citations. CMNPD aims to provide an open access knowledge base for not only professional MNPs researchers but also the broader scientific community to facilitate the research and development of marine drugs.

## DATA CONTENT

### Data extraction and curation

Compound records were mainly extracted from the remarkable annual MNPs reviews published by the late D. John Faulkner in *Nat. Prod. Rep*. (11), a series continued by the team of John W. Blunt (12), and now Anthony R. Carroll (13). The cited references list was retrieved through Web of Science (https://apps.webofknowledge.com, 2018) and then imported into EndNote (version 9.0.0, Clarivate Analytics Inc. 2018) to obtain full text. After manual curation, more than 20 000 articles covering the period from the1960s to December 2018 were collected and integrated into a main document library. Most of these articles focused on the reports of new compounds and the relevant biological activities, together with previously reported compounds where there had been a structural revision or a newly established stereochemistry. The chemical structure, compound name, source organism and other information of small molecules were manually extracted and curated from these publications. The structure of MNPs was then used as a query to search in generic chemical databases (e.g. Reaxys, PubChem, ChEMBL), and the scientific literature and patents of hit compounds were integrated into a general document library.

In order to improve the efficiency of structure extraction, the optical chemical structure recognition tool CLiDE (version 5.12.1, Keymodule Inc. 2017) was used to convert the graphical representations of chemical structures to machine-readable format and transfer them into the chemical editor ChemDraw (version 19.1, PerkinElmer Inc. 2020) for manual inspection and correction. The conformation of the chemical structure was kept consistent with the molecular image in the literature as far as possible to improve recognizability. The chair conformation, Haworth projection and Fischer projection were converted to wedge-dash diagrams to ensure that the stereochemistry could be recognized correctly by the computer software and the 3D shapes could be accurately depicted on the 2D screen. When substituent group abbreviations were expanded, the angle and length of some bonds were adjusted to prevent stacking. The 2D structures were saved in MOL format and then converted into multiple formats (e.g. SMILES, InChI, InChIKey) via Pipeline Pilot (version 18.1, BIOVIA Inc. 2018). Each structure was classified into its corresponding chemical classes using the ClassyFire web server (14). NMR, IR, Raman, UV-Vis, and mass spectra of the structures were presented if available from Wiley SpectraBase (https://spectrabase.com, 2020).

The 3D conformers were generated using OMEGA (version 3.0.0.1, OpenEye Scientific Software Inc. 2018). When undefined stereocenters existed, each stereoisomer was enumerated and conformers independently generated. A maximum of 100 000 conformers per stereoisomer were allowed, and the lowest-energy conformer of each stereoisomer was retained at last. Since a certain number of complicated MNPs contain many undefined stereocenters and/or rotatable bonds, it makes no sense to compute 3D descriptions for all records. Therefore, according to the criteria of PubChem3D conformer models, CMNPD provides a 3D representation for each compound that satisfies the following conditions: (i) not too large (with no more than 50 heavy atoms), (ii) not too flexible (with no more than 15 rotatable bonds), (iii) has only a single covalent unit (salt, mixture or polymer keep only the largest fragment in the calculation), (iv) consists of only supported elements (H, C, N, O, F, Si, P, S, Cl, Br and I), (v) contains only atom types recognized by the MMFF94s force field, (vi) has fewer than six undefined atom or bond stereocenters (15). As a result, 79.9% of all records have 3D information.

### Calculated properties

To estimate the drug-likeness of each compound, some physicochemical and pharmacokinetic properties were calculated using widely accepted algorithms. Physicochemical properties were calculated using RDKit (https://www.rdkit.org, 2020), including molecular weight, molecular mass (i.e. monoisotopic mass), octanol/water partition coefficient ALogP (16), polar surface area (17) and quantitative estimate of drug-likeness QED weighted (18), as well as the number of rotatable bonds, hydrogen bond acceptors, hydrogen bond donors, aromatic rings and heavy atoms. Predictions of pharmacokinetic properties were calculated using Pipeline Pilot ADMET models, including blood brain barrier penetration, human intestinal absorption (19,20), aqueous solubility (21), CYP2D6 binding (22), hepatotoxicity (23) and plasma protein binding (24).

### Organism resources

Isolation from the source marine organism is the only way to obtain a promising MNP if it cannot be synthesized. Therefore, it is necessary to record what organisms this compound was isolated from and where these organisms were collected, which is also critical to guarantee the reproducibility of relevant research and maximize the bioprospecting efficiency of MNPs (25). Taxonomic information and sampling location of source organisms were extracted from the articles in the main document library, some of which referred to the authors’ previous papers. All organisms were classified into seven hierarchies (i.e. kingdom, phylum, class, order, family, genus and species) based on the authoritative taxonomic databases, such as the Catalogue of Life (CoL, version 2019 annual checklist, http://www.catalogueoflife.org, 2020), the World Register of Marine Species (WoRMS, http://www.marinespecies.org, 2020), the Integrated Taxonomic Information System (ITIS, https://www.itis.gov, 2020) and the Index Fungorum (http://www.indexfungorum.org, 2020). The taxonomic names of identified species were normalized to accepted scientific names, and the unaccepted names (e.g. original combination, new combination, replacement name, incorrect spelling) were recorded as synonymised names. Some of the (newly) identified species that had not been published with an adequate taxonomic description were not included in the above taxonomic databases. Hence, their names were marked as *nomina nuda* (naked names) temporarily. Sampling locations were converted to coordinates using Google Maps (https://www.google.com/maps, 2020). Some articles and their cross references did not describe where the organisms were collected. As a stopgap measure, the address of the author’s affiliation was regarded as the resource location.

### Biological activity data

A certain amount of biological activity data based on the initial bioassays of the discoverers was extracted from the main document library, but most of it was brief description of the pharmacological effects, such as ‘cytotoxic’, ‘antibacterial’, ‘antifungal’ and ‘anti-inflammatory’. As one can imagine, substantial bioactivity data is deposited in the generic chemical databases, especially those that store medicinal chemistry data. To capture the greatest quantity of high-quality bioactivity data efficiently, assay and bioactivity information from the ChEMBL database (release 27, 2020), which exchanges data with dozens of datasets such as PubChem BioAssay (26) and BindingDB (27), were incorporated into the CMNPD standardized experimental dataset. This detailed dataset includes target name, target type (e.g. nucleic acid, protein, cell line, tissue, organism), target organism, activity type (e.g. IC50, Ki, ED50, EC50, mortality), activity value, assay type (e.g. binding, functional, ADME, toxicity, physicochemical) and assay description. To provide more authoritative information about the targets, proteins were mapped into the Universal Protein Resource (UniProt) (28), the Protein Data Bank in Europe (PDBe) (29), the Gene Ontology Annotation (GOA) resource (30), the Therapeutic Target Database (TTD) (31) and the Open Targets Platform (32), while cell lines were mapped into the the Cell Line Ontology (CLO) (33), the Cell Ontology (CL) (34), the Experimental Factor Ontology (EFO) (35), the Cellosaurus (36) and the Library of Integrated Network-based Cellular Signatures (LINCS) NIH program (37).

### Current content

**Figure 1.**
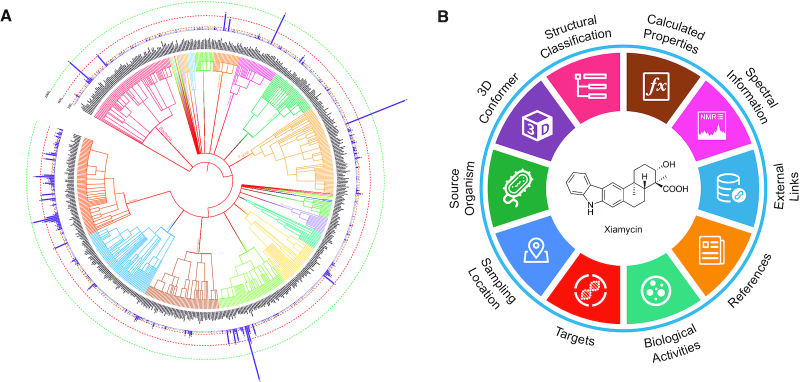
Schematic overview of the CMNPD content. (**A**) Phylogenetic tree of marine organisms. Core nodes include domains, kingdoms, phyla, classes, orders and families. Internal nodes include their corresponding superior and subordinate taxonomic units, which are based on the NCBI Taxonomy database (38). The bar chart shows the number of compounds in each family. This figure was produced using the Interactive Tree Of Life (iTOL) web server (39). (**B**) Knowledge graph of the chemical entity.

The content of CMNPD is demonstrated in Figure 1 and the statistical data is summarized in Table 1. In release 1.0, CMNPD contains 31 561 distinct chemical entities of MNPs from over 13 000 sampling organisms. These organisms are distributed in 7 kingdoms, 38 phyla, 93 classes, 289 orders, 682 families, 1480 genera and 3354 species. There are 15 774 active compounds mapped to 2652 targets with 72 343 bioactivities. These targets include 1122 single proteins, 923 cell lines, 459 organisms and several other types. The document library includes 128 488 scientific literature and patents, of which ∼11 000 articles describe the discovery of new compounds and structure revisions.

**Table 1.** Statistics of various data collections in CMNPD (release 1.0)

Data collection	Count	Description
**Compounds**		Marine natural products
Chemical entities	31 561^a^	Unique chemical structures
Compounds with 3D conformers	25 224	Filter according to the criteria of PubChem3D conformer models
Biologically active compounds	15 774	Compounds with biological activity data
**Organisms**		Source marine organisms
Kingdoms	7	Taxonomic hierarchy
Phyla	38	Taxonomic hierarchy
Classes	93	Taxonomic hierarchy
Orders	289	Taxonomic hierarchy
Families	682	Taxonomic hierarchy
Genera	1480	Taxonomic hierarchy
Species	3354^b^	Taxonomic hierarchy
**Targets**	2652	Targets standardized according to ChEMBL target list
Single proteins	1122	Target type
Cell lines	923	Target type
Organisms	459	Target type
Others	148	Target type
**Bioactivities**	72 343	Biological activity data
Data in brief	15 980	Manual collection from literature
Standardized experimental data	56 363	Incorporation from ChEMBL
**Documents**	128 488	Scientific literature and patents
Literature	119 543	Literature abstracts/citations
Patents	8945	Patent abstracts/citations

^a^Chemical entities include different forms of certain compounds (e.g. original structure, revised structure, stereochemically improved structure, controversial structure).

^b^This is the minimum estimate of the species count, because many unidentified species have been classified into the same category (e.g. *Sinularia* sp., unidentified species of Family Spongiidae).

## DATA ACCESS

### Web interface

CMNPD could be accessed at https://www.cmnpd.org with a user-friendly interface. This interface is modelled on the new web interface of ChEMBL (9) to enhance user experience and reduce learning costs. It allows users to browse, search, and explore MNPs-related information in a variety of ways.

#### Data browsing

Four main types of entries (compounds, organisms, targets and documents) assigned with unique CMNPD identifiers could be browsed on the full list pages and the dedicated report card pages. The full list pages provide interactive filters that can be applied to show the distribution of the dataset with regard to several specific properties (e.g. molecular weight, target type, organism hierarchy), and to allow users to browse a subset of the original data in a given range for the filter property. The report card pages provide further details about the entries, such as name and classification (for compounds, organisms and targets), literature/patent bibliographic information (for documents), structure, calculated properties and biological activities (for compounds), together with internal links to other report card pages and external links to other resources (e.g. PubChem, UniProt, Catalogue of Life).

#### Data query

**Figure 2.**
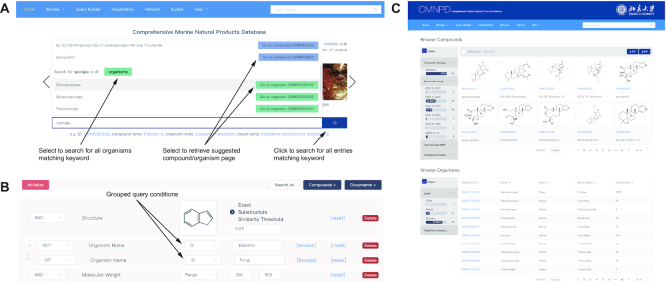
Data query in the web interface. (**A**) Free-text quick search. Users can retrieve a list of entities matching a selected keyword (e.g. all organisms matching ‘spongia’) or go directly to a report card page for a selected entry (e.g. organism Demospongiae: CMNPD330032). (**B**) Advanced search. Query conditions are combined with Boolean operators. The inner Boolean operation of the grouped conditions is executed first, and then the outer Boolean operations are performed in order from top to bottom. (**C**) Search result of the keyword ‘spongia’. Users can further select the entries through the interactive filters on the left.

Quick search is available in the middle of the homepage and on the toolbar in the upper right corner of each page. The free-text search allows users to enter any term of CMNPD identifier, compound name, organism name or target name without specifying a search entity. The search bar will provide suitable suggestions as the term is typed (Figure 2A).

In addition, a powerful advanced search capability is provided on the query builder page. This allows users to specify any number of query conditions. Available query conditions, which could be combined with the Boolean operator ‘AND’, ‘OR’ or ‘NOT’, include structure (drawing structure, structural classification), compound representations (e.g. compound name, molecular formula), physicochemical properties (e.g. molecular weight, ALogP), ADMET prediction (e.g. blood brain barrier penetration level, human intestinal absorption level), resources (organism name, collection site), bioactivities (e.g. target name, assay type) and bibliography (e.g. authors, DOI). Multiple query conditions could be easily grouped together by just dragging and dropping them. The inner Boolean operations of the grouped conditions will be executed first (Figure 2B). The structure search module, entered with a drawing or a SMILES or InChI string using Marvin JS sketcher (version 19.9.0, ChemAxon Ltd. 2019), allows users to select among exact match, substructure and similarity. Chemical similarity is computed with the Morgan fingerprints and the Tanimoto metric. These fingerprints are generated using RDKit. Some query conditions (e.g. organism name, target name) have both text entry and browse functions, so users have the choice of typing in a search term or selecting from the index.

#### Data visualizations

**Figure 3.**
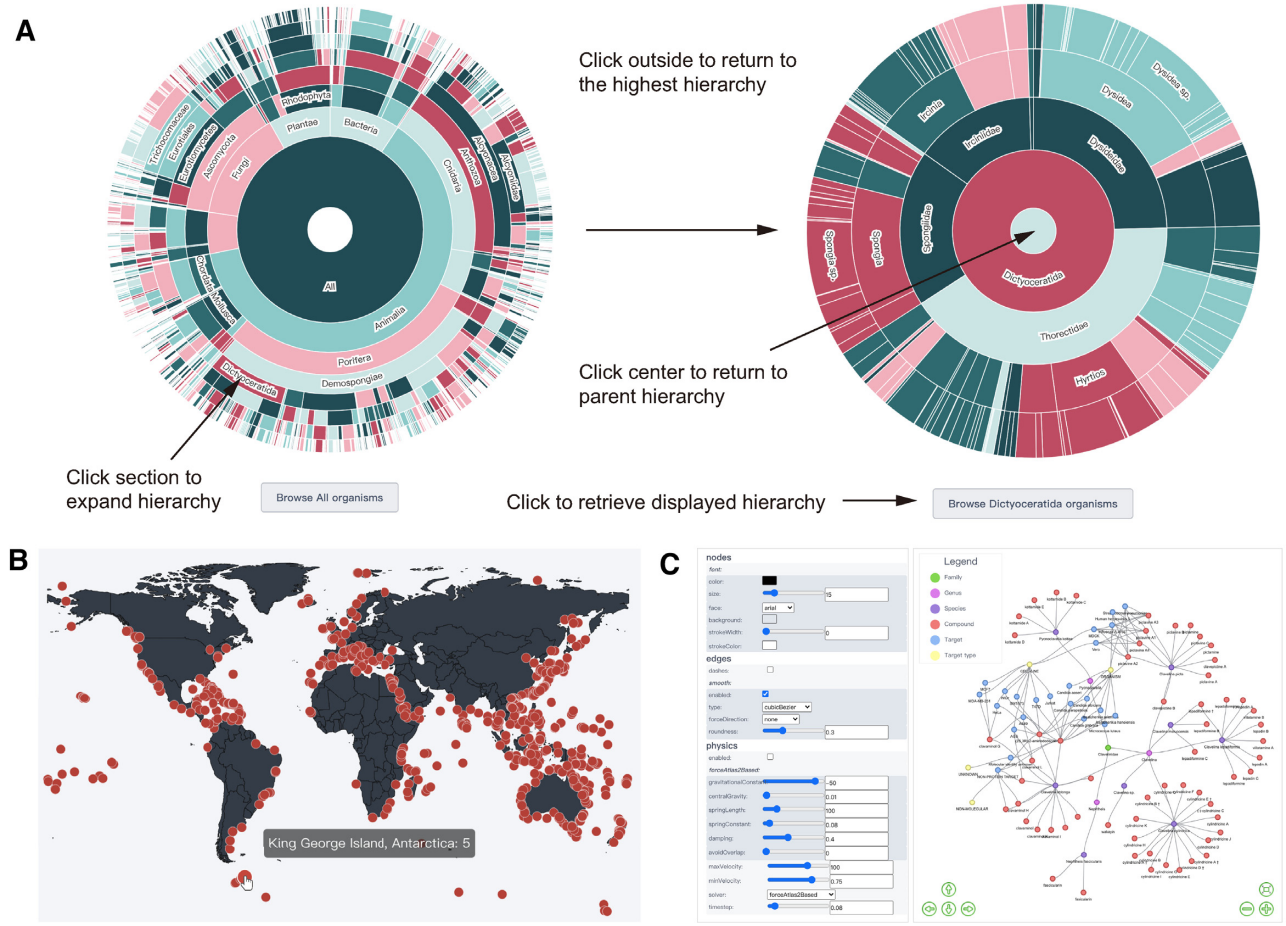
Data visualizations. (**A**) Sunburst chart showing different hierarchies of organism taxonomy. The classification is arranged in concentric circles, and from inside to outside are kingdoms, phyla, classes, orders, families, genera and species. Users can click a section to expand the hierarchy and the corresponding entries can be retrieved using the button below the chart. (**B**) Dot distribution map showing the collection sites of organisms (e.g. sponges). Users can click the dot on the map to retrieve the compounds discovered in the corresponding area. (**C**) Network showing the relationship between organisms, compounds and targets. Users can click any node to enter the report card page of the corresponding entry.

To help users better understand and explore the data inside CMNPD, several interactive data visualizations have been created with the JavaScript graphing libraries Plotly (https://plotly.com/javascript, 2020) and ECharts (version 4.0, https://echarts.apache.org, 2020). The sunburst charts demonstrate different hierarchies of organism taxonomy and structural classification. Any section can be expanded by clicking and the corresponding entries can be retrieved using the button below the chart (Figure 3A). The dot distribution map shows the organism collection or storage sites of each compound. Clicking the dot on the map can retrieve the compounds discovered in the corresponding area. The distribution map is also available on each organism report card page and compound report card page (Figure 3B).

To intuitively illustrate the relationship between organisms, compounds and targets, a systematic analysis function is provided using vis-network (version 7.7.0, https://visjs.org, 2020), which allows users to build network visualization by selecting a master node from these entries. Clicking on the node can retrieve the detailed information of the corresponding entry (Figure 3C).

### Downloads

Some users would like to download the database for data mining or virtual screening besides querying it via the web interface. Bulk downloads of several complete datasets are available at https://docs.cmnpd.org/downloads. In addition, users can customize the compound download list through the advanced search and manual selection.

## DATA DEPOSITION

To improve the quantity and quality of data, CMNPD provides a deposit system, which allows users to submit new compounds, new data of existing compounds and corrections to existing data. Only the published data is acceptable, and references must be attached at the time of submission. Scholars engaged in MNPs research are welcome to submit new compounds to CMNPD once the paper is accepted by the journal. When depositing a new chemical entity, the MOL format is preferable to SMILES or InChI strings, which ensures that the conformational structure expression is consistent with that in the publication.

## SUMMARY

Marine organisms are regarded as an important source of inspiration for drug discovery after terrestrial plants and microorganisms. Half of the discovered MNPs have various biological activities. Several marine-derived drugs (e.g. Ziconotide, Trabectedin, Eribulin) have been approved by FDA, and more candidates are in clinical trials (40). The fact that there are few approved marine-derived drugs is certainly not due to the limited chemical diversity of MNPs. Actually, material supply issues remain the major obstacle to marine drug discovery. In the absence of universality for total synthesis and mariculture, information sharing is particularly important for the development of MNPs research.

To make the best of the full potential offered by the chemical diversity of the secondary metabolites from marine organisms for drug discovery, we present CMNPD as an open access knowledge base with comprehensive data, intuitive web interface and advanced retrieval system for the broad scientific community. CMNPD supplies accurate chemical structures and various calculated physicochemical and pharmacokinetic properties for computer-aided drug design as well as detailed taxonomic and geographic information of source organisms for the study of chemical ecology. The standardized experimental dataset integrates the ChEMBL database to provide high-quality biological activity data. In future, we expect CMNPD to grow continuously with extensive data deposition and resource integration, becoming an even more comprehensive MNPs repository that could lead the wave of marine drug development.

## DATA AVAILABILITY

CMNPD is made available under a Creative Commons Attribution-NonCommercial-ShareAlike 4.0 International license (https://creativecommons.org/licenses/by-nc-sa/4.0).
